# Supplementary material for: The scaffold protein PRR14L links the PP2A-TACC3 axis to mitotic fidelity and sensitivity to MPS1 inhibition
Source: bioRxiv. 2025 Dec 29:2025.11.04.686150. Originally published 2025 Nov 6. Preprint. [Version 2] doi: 10.1101/2025.11.04.686150 (PMC12637489; doi:10.1101/2025.11.04.686150)

# Supplemental Figure 1

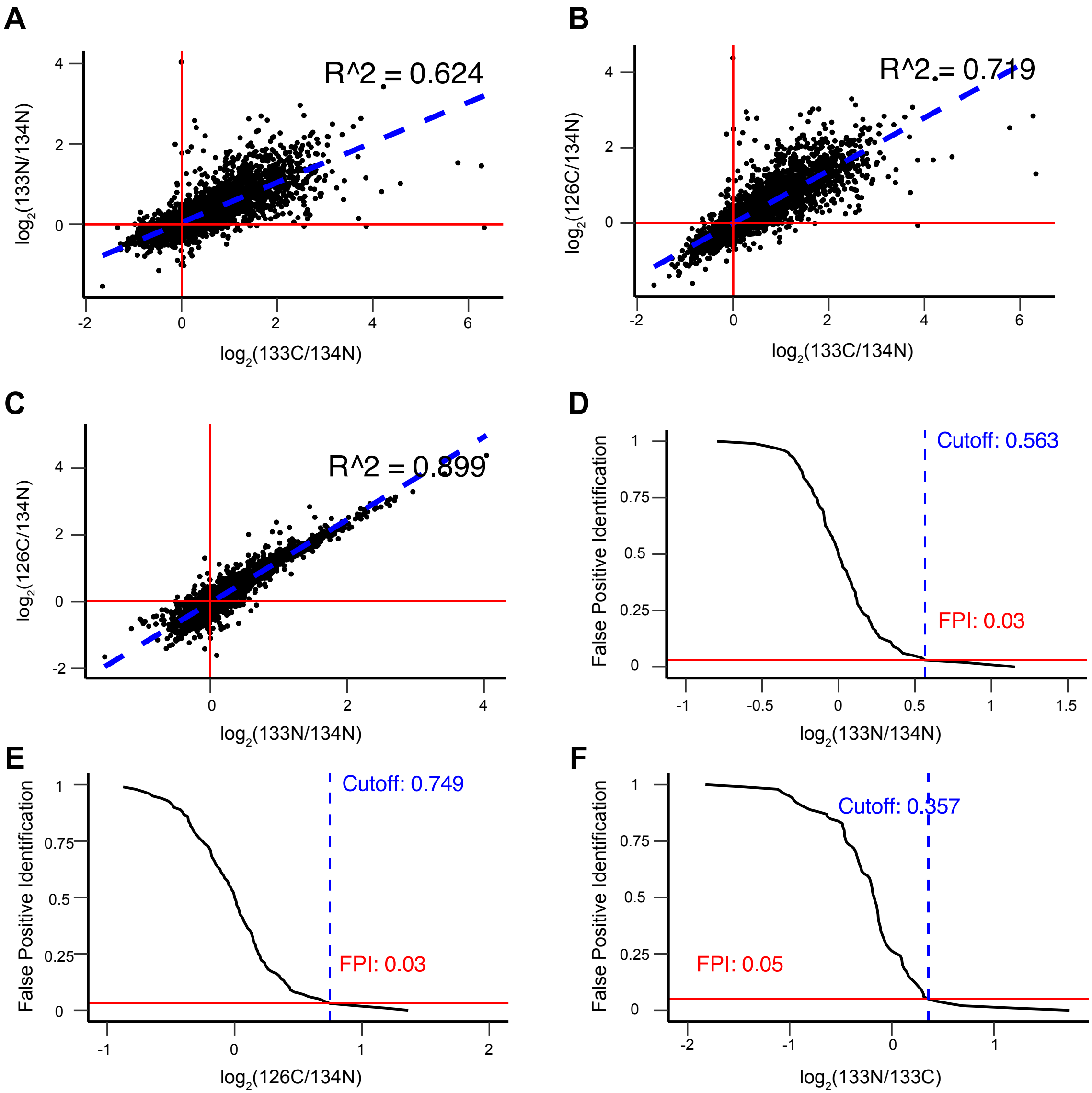

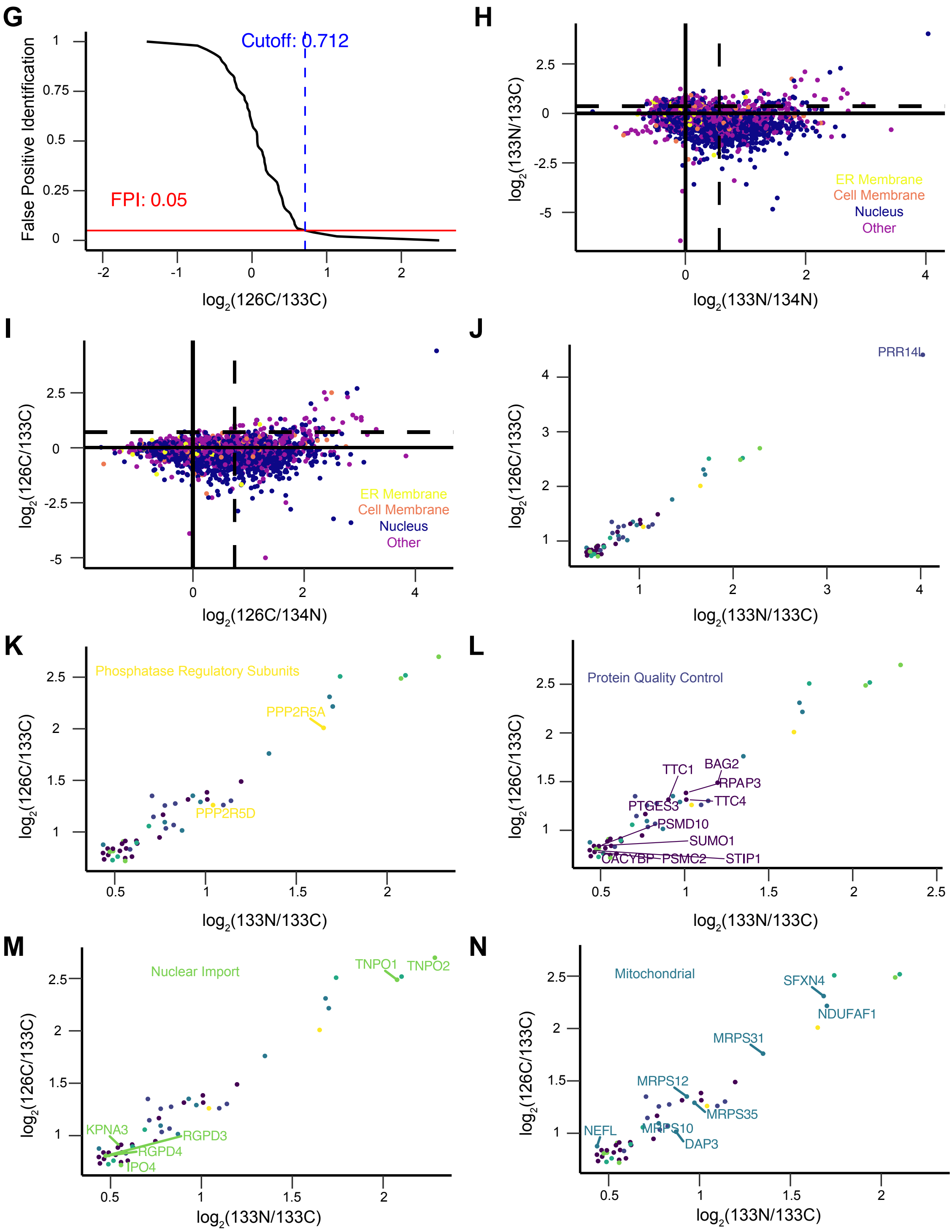

G
False Positive Identification
log2(126C/133C)
Cutoff: 0.712
FPI: 0.05
H
log2(133N/134N)
log2(133N/133C)
ER Membrane
Cell Membrane
Nucleus
Other
I
log2(126C/134N)
log2(126C/133C)
ER Membrane
Cell Membrane
Nucleus
Other
J
log2(133N/133C)
log2(126C/133C)
PRR14L
K
Phosphatase Regulatory Subunits
PPP2R5A
PPP2R5D
log2(133N/133C)
log2(126C/133C)
L
Protein Quality Control
TTC1
BAG2
RPAP3
TTC4
PTGES3
PSMD10
SUMO1
CACYBP
PSMC2
STIP1
log2(133N/133C)
log2(126C/133C)
M
Nuclear Import
TNPO1
TNPO2
KPNA3
RGPD3
RGPD4
IPO4
log2(133N/133C)
log2(126C/133C)
N
Mitochondrial
SFXN4
NDUFAF1
MRPS31
MRPS12
MRPS35
NEFL
MRPS10
DAP3
log2(133N/133C)
log2(126C/133C)

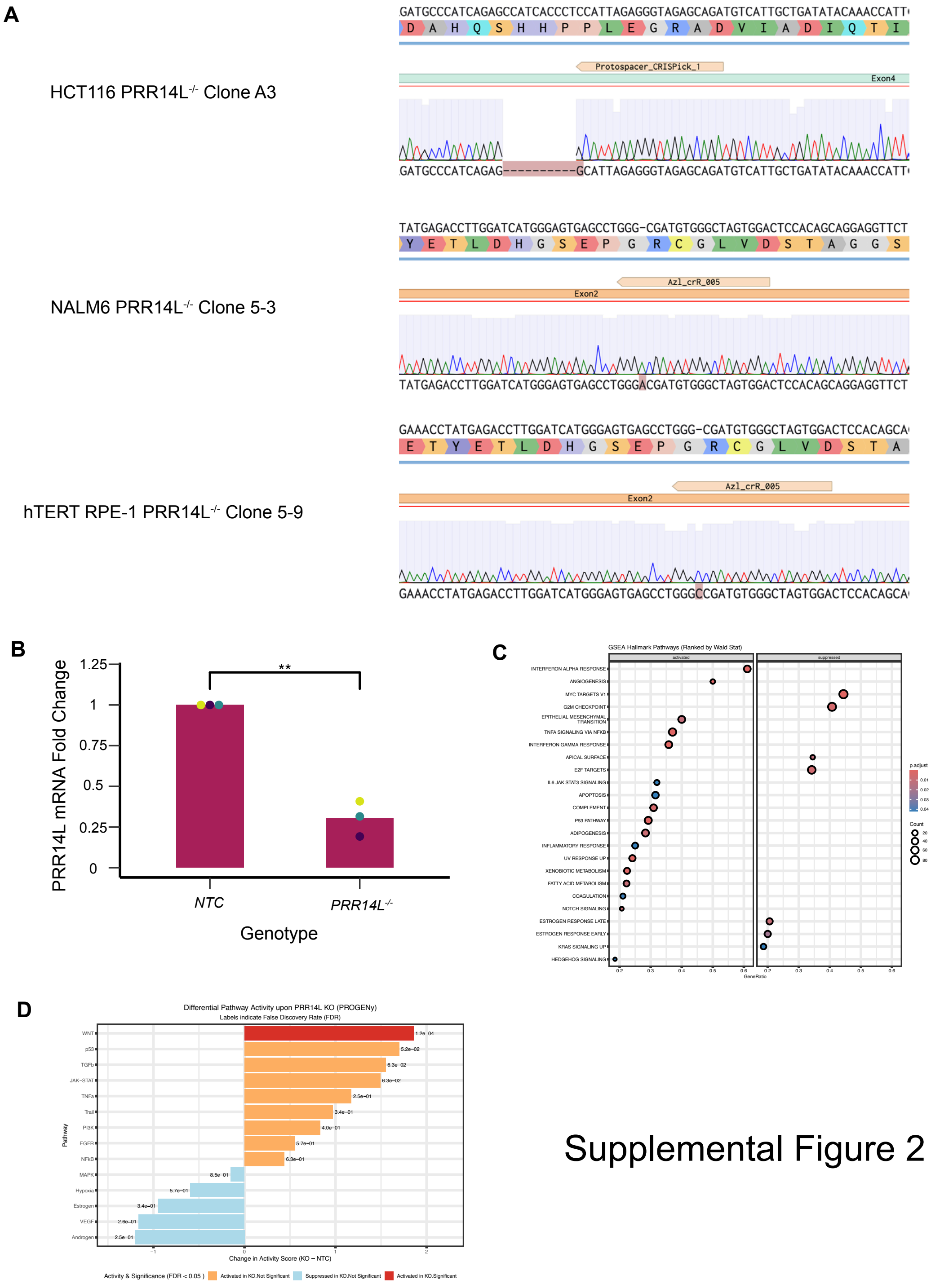


Supplemental Figure 2

# Supplemental Figure 3

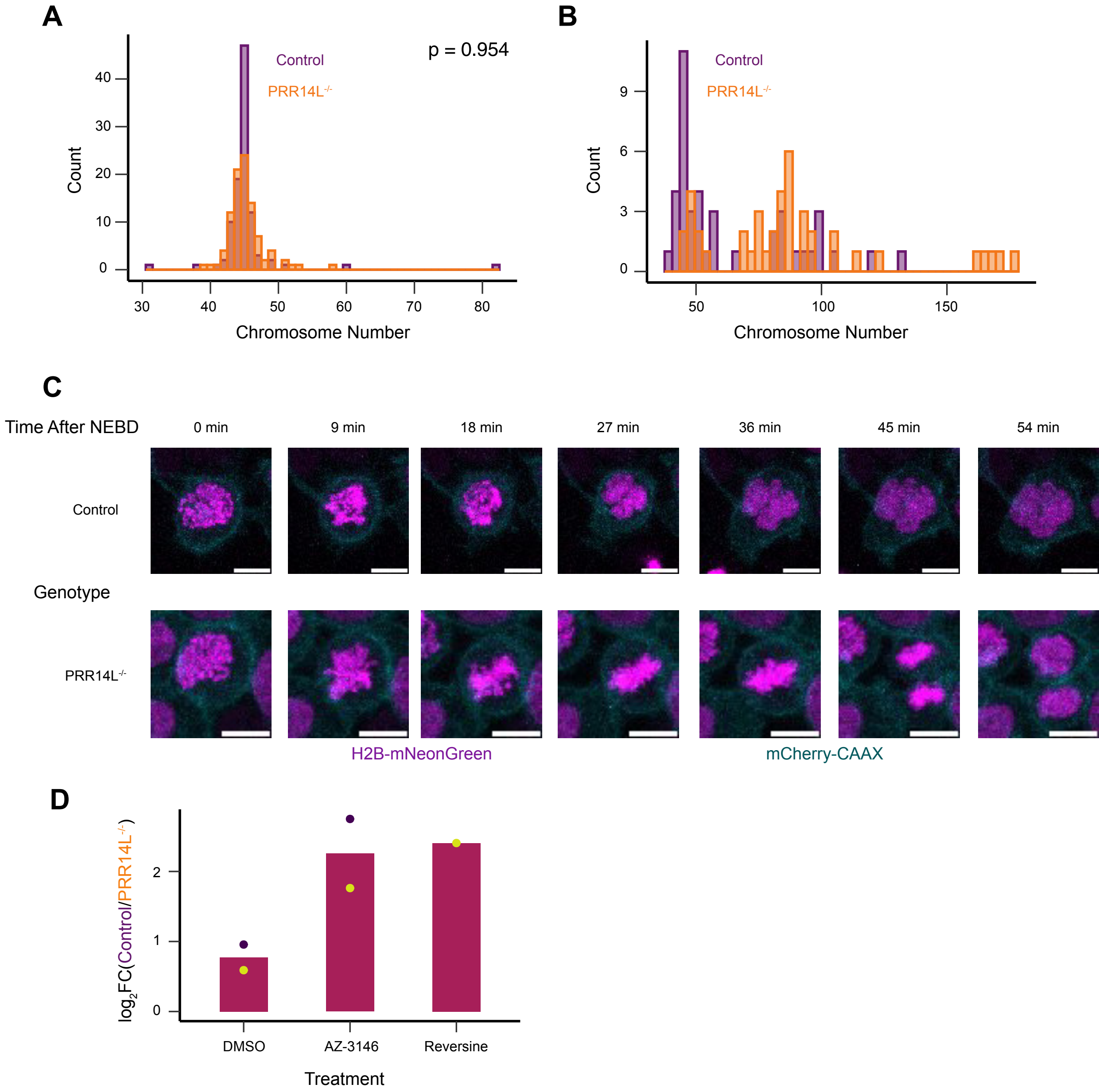

Supplement: Supplement 1 — Figure S1. (A)Linear correlation between PRR14L-TurboID independent experiment 1 and TurboID-NLS. Scatter plot displaying all proteins with >2 unique peptides identified by mass spec, comparing replicate 1 against TurboID-NLS. (B)Linear correlation between PRR14L-TurboID independent experiment 2 and TurboID-NLS. Scatter plot displaying all proteins with >2 unique peptides identified by mass spec, comparing replicate 2 against TurboID-NLS. (C)Linear correlation between PRR14L-TurboID independent experiment 1 and independent experiment 2. Scatter plot displaying all proteins with >2 unique peptides identified by mass spec, comparing replicate 2 against replicate 1. (D)Determination of filter 1 cut-off for independent experiment 1. False positive protein list used was ER membrane proteins from Uniprot. False positive identification plotted against log2 ratio comparing independent experiment 1 and no ligase control. Log2 cut-off determined at a FPI = 0.03 (3% of false positive proteins have log2 ratios higher than the cut-off of 0.592). (E)Determination of filter 1 cut-off for independent experiment 2. False positive protein list used was ER membrane proteins from Uniprot. False positive identification plotted against log2 ratio comparing independent experiment 2 and no ligase control. Log2 cut-off determined at a FPI = 0.03 (3% of false positive proteins have log2 ratios higher than the cut-off of 0.804). (F)Determination of filter 2 cut-off for independent experiment 1. False positive protein list used was cell membrane proteins from Uniprot. False positive identification plotted against log2 ratio comparing independent experiment 1 and spatial control. Log2 cut-off determined at a FPI = 0.05 (5% of false positive proteins have log2 ratios higher than the cut-off of 0.335). (G)Determination of filter 2 cut-off for independent experiment 2. False positive protein list used was cell membrane proteins from Uniprot. False positive identification plotted against log2 ratio [file media-1.pdf]
